# Lipid Abundance and Metabolism Aberrations in Skin Diseases

**DOI:** 10.3390/metabo14110627

**Published:** 2024-11-15

**Authors:** Julia Nowowiejska

**Affiliations:** Department of Dermatology and Venereology, Medical University of Bialystok, 15-089 Bialystok, Poland; julia.nowowiejska@umb.edu.pl

This Special Issue of *Metabolites*, entitled “Lipid Abundance and Metabolism Aberrations in Skin Diseases” broadly discusses the issue of lipids that are engaged in pathogenesis, as well as in the clinical course and treatment of dermatoses.

The authors participating in this Special Issue primarily concentrate on psoriasis, which is one of the most common chronic skin diseases, as well as on lichen planus and even rarer diseases, such as porokeratosis.

In the field of psoriasis, several advancements have been made. Matwiejuk et al. comprehensively discussed the role of sphingolipids in psoriasis [1], pointing out the important role of altered levels of ceramides in psoriatic skin, which lead to anti-apoptotic and pro-proliferative conditions, the hyperproliferation of keratinocytes, and the development of skin lesions. Psoriatic patients with higher S1P serum concentrations may be predisposed to the development of metabolic syndrome [1]. In their second paper, they review the influence of hypolipidemic medications on psoriasis [2]. In the majority of reported cases, the positive role of statins, fibrates, glitazones, and analogs of GLP-1 in the additional treatment of psoriasis has been noted. The reduction in psoriasis severity and an increased quality of life have been observed. On the other hand, some cases of psoriasis exacerbation have been reported as a result of the same drugs [2]. 

In the field of lichen planus, our team aimed to analyze the lipid disturbances in this type of dermatosis [3]. The available evidence indicates that dyslipidemia is an important problem in patients with this dermatosis, particularly hypertriglyceridemia. Dermatologists should pay attention to this relationship and be aware of the different metabolic complications. Perhaps simple laboratory investigations of lipid parameters in such patients or measurements of their waist circumference in daily dermatological practice could benefit these patients; however, more studies are required to establish guidelines regarding such approaches [3]. Ilves et al. also focused on lichen planus [4]; their experiment proved the altered composition of lipoproteins in the serum of patients with lichen planus. The composition of lipoproteins affected their function; therefore, the authors suggested that the detected changes may place patients with LP in the at-risk category of metabolic syndrome, dyslipidemia, and diabetes mellitus type 2. They also highlighted the need for screening for dyslipidemia in patients with LP [4].

Pietkiewicz et al. concentrated on porokeratosis, which is a vast, but rare, group of skin disorders characterized by the presence of cornoid lamella [5]. Their team covered the genetic background of porokeratosis, as well as metabolomic profiles and imaging methods—including dermoscopy, confocal microscopy, and pathology. They highlighted the fact that due to the risk of cancer, patients should be monitored in the long term.

To conclude, the analysis of lipid components in various dermatological diseases may serve as a worthy tool in the discovery of pathogenesis gaps, the identification of new disease variants, the prediction of the dermatosis course or complications, and, finally, to provide information for personalized treatment.
